# Deep learning-assisted near-real-time identification of the inferior mesenteric artery and vein during laparoscopic rectal resection: a single-center proof-of-concept study

**DOI:** 10.3389/fmed.2026.1891914

**Published:** 2026-08-21

**Authors:** Yan Gao, Di Hao, Yu Yang, Han-Hui Jing, Zong-Sheng Sun, Xiao-Dong Liu, Zheng Jiang, Dong-Rui Li, Hai-Qiang Zhang, Wen-Feng Feng, Gang Liu, Guang-Ye Tian, Shang-Long Liu

**Affiliations:** 1 Department of Gastrointestinal Surgery, The Affiliated Hospital of Qingdao University, Qingdao, Shandong, China; 2 School of Control Science and Engineering, Shandong University, Jinan, Shandong, China; 3 Department of Colorectal Surgery, National Cancer Center/Cancer Hospital, Chinese Academy of Medical Sciences, Beijing, China; 4 Department of General Surgery, The Second Hospital of Hebei Medical University, Shijiazhuang, Hebei, China; 5 Department of General Surgery, Tianjin Medical University General Hospital, Tianjin, China

**Keywords:** artificial intelligence, computer vision, deep learning, laparoscopic surgery, near-real-time navigation, semantic segmentation, vessel recognition

## Abstract

**Background:**

Reliable identification of the inferior mesenteric artery (IMA) and inferior mesenteric vein (IMV) is essential for safe vascular control during laparoscopic rectal resection (LRR). We developed and internally evaluated a near-real-time semantic segmentation model using standard white-light laparoscopic video to support intraoperative anatomical recognition.

**Methods:**

This single-center retrospective proof-of-concept study included operative videos from 16 patients who underwent LRR between February 2024 and May 2025. Frames were sampled at 1 frame/s, yielding 2,720 expert-annotated RGB images (1,758 IMA images and 962 IMV images). Data were divided at the patient level into a training/development cohort (13 patients, 2,260 frames) and an internal holdout test cohort (3 patients, 460 frames). A frame-wise two-dimensional U-Net was configured using nnU-Net v2. Multiclass segmentation was compared with vessel-specific binary segmentation. Twenty gastrointestinal surgeons who had not participated in model development assessed preliminary usability and acceptance using three 0–4 Likert items.

**Results:**

Vessel-specific binary segmentation produced more balanced performance, with the clearest improvement for the IMV. In the internal holdout test cohort, the IMA Dice coefficient, precision, and recall were 0.940 +/− 0.025, 0.945 +/− 0.023, and 0.940 +/− 0.020, respectively; the corresponding IMV values were 0.980 +/− 0.010, 0.982 +/− 0.017, and 0.978 +/− 0.018. On an NVIDIA RTX 3090 GPU, inference reached 12.7 frames/s, with approximately 0.08 s of network processing per frame. Surgeons rated perceived recognition accuracy at 3.41 +/− 0.09 and future clinical potential at 3.39 +/− 0.09 on the 0–4 scale.

**Conclusion:**

The nnU-Net-configured two-dimensional U-Net achieved high segmentation scores on selected internal test data and supported near-real-time visualization. These findings provide early evidence of technical feasibility rather than clinical effectiveness. Multicenter external validation, testing in difficult operative fields, objective human-factors experiments, and prospective clinical evaluation are required before clinical use.

## Introduction

Total mesorectal excision (TME) is the oncological foundation of curative rectal cancer surgery. Laparoscopic rectal resection (LRR) is widely performed and can provide oncological outcomes comparable to open surgery while preserving the benefits of a minimally invasive approach (1–6). Nevertheless, vascular injury and unexpected bleeding remain clinically important hazards. The confined pelvic workspace, continuously changing camera perspective, absence of tactile feedback, and overlap among fat, fascia, vessels, autonomic nerves, and ureters make intraoperative recognition strongly dependent on surgical experience and an accurate understanding of embryological planes (7–13).

The inferior mesenteric artery (IMA), its branches, and the inferior mesenteric vein (IMV) are major landmarks during medial-to-lateral mobilization. Early recognition of the IMA origin helps define the lymphadenectomy field and the correct dissection plane, whereas identification of the IMV supports safe mobilization of the left mesocolon and vascular division. Preoperative computed tomography angiography can depict vascular variants, but it provides a static map that does not fully account for displacement caused by pneumoperitoneum, patient positioning, traction, and progressive tissue mobilization (10, 11, 14). A system that updates with the current laparoscopic view may therefore complement, rather than replace, preoperative imaging and surgeon judgment.

Computer vision has increasingly been applied to surgical instrument detection, phase recognition, and the identification or segmentation of nerves, organs, loose connective tissue, and safe dissection planes (15–26). For vascular navigation, semantic segmentation offers pixel-level contours rather than rectangular bounding boxes. This distinction is important because vessels are slender, branching, and often only partly exposed; their course and wall boundaries may directly influence the route of dissection. Previous studies have explored automated recognition of the IMA and right-sided colonic vessels, but the evidence remains dominated by small, single-center feasibility studies (27, 28).

Pixel-level overlays may show vessel direction, relationships to the mesenteric edge, and potential branches more effectively than object-detection boxes. However, inaccurate or unstable overlays may distract the surgeon. Evaluation should therefore extend beyond spatial overlap to latency, failure modes, user comprehension, and suppression of output when confidence is low. In this study, we developed an nnU-Net-based model for near-real-time IMA and IMV segmentation and evaluated frame-level performance, multiclass versus vessel-specific binary labeling, inference efficiency, and preliminary surgeon acceptance. Reporting was guided by the principles of DECIDE-AI for early-stage evaluation of artificial intelligence dxecision-support systems (29). The completed DECIDE-AI reporting checklist is provided as Supplementary Data Sheet 1.

## Methods

### Study design, participants, and data partitioning

This single-center retrospective proof-of-concept study was conducted in the Department of Gastrointestinal Surgery at The Affiliated Hospital of Qingdao University. Operative videos from 16 patients who underwent LRR between February 2024 and May 2025 were included. The institutional ethics committee approved the protocol (QYFYWZLL30382). All videos were de-identified before analysis, and the requirement for informed consent was waived because only anonymized retrospective data were used.

Eligibility required: (1) a complete laparoscopic TME recording; (2) a video sequence in which exposure and division of the IMA and/or IMV could be followed; and (3) confirmation of vessel identity by at least two gastrointestinal surgeons. Exclusion criteria were missing or corrupted video files, persistently inadequate image quality for reliable annotation, a principal operative approach other than laparoscopic TME, markedly altered anatomy from previous rectal surgery, or conversion to open surgery.

**FIGURE 1:**
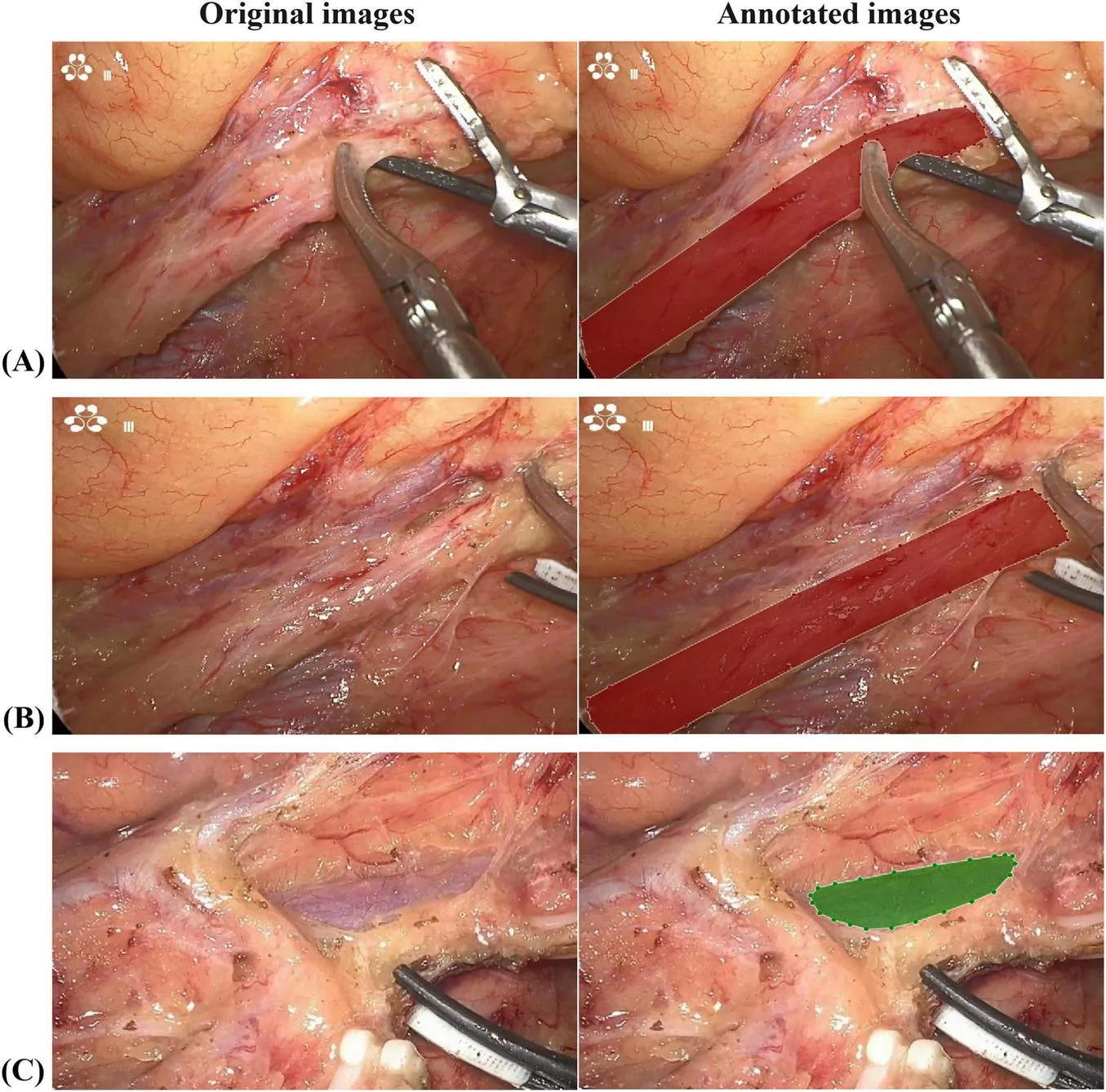
Representative images from the dataset. The left panels show the original images, and the right panels show the corresponding annotated images: **(A)** annotation of the IMA when partially occluded by surgical instruments; **(B)** annotation of the IMA when exposed; and **(C)** annotation of the IMV when exposed.

The 16 procedures yielded 2,720 annotated images, including 1,758 IMA images and 962 IMV images; 108–228 frames were selected from each case. To reduce leakage caused by the high similarity of adjacent frames from the same operation, all partitioning was performed at the patient level. The training/development cohort comprised 13 patients and 2,260 frames, whereas the independent internal holdout test cohort comprised 3 patients and 460 frames. Patient-level five-fold cross-validation within the training/development cohort was used for model selection, and no patient contributed images to more than one subset. Representative original and annotated vessel images are shown in Figure 1.

### Video preprocessing and image sampling

All recordings were converted to MP4 format at a resolution of 1,920 × 1,080 pixels and an original frame rate of 30 frames/s. Key clips were extracted from the beginning of IMA-root exposure through completion of IMA and IMV division. RGB frames were then sampled at 1 frame/s and analyzed as independent two-dimensional images. The exported nnU-Net configuration used two-dimensional 3 × 3 convolutions and a sliding-window patch size of 896 × 1,792 pixels; consequently, the current implementation did not explicitly model temporal continuity between frames.

Channel-specific preprocessing parameters were learned automatically from the training/development data and applied unchanged during training and inference. Red-channel intensities were clipped to 72–255 and standardized using a mean of 163.3 and standard deviation of 40.7. Green-channel intensities were clipped to 28–244 and standardized using 104.5 and 41.1, respectively. Blue-channel intensities were clipped to 23–231 and standardized using 88.9 and 38.4, respectively (Table 1).

**TABLE 1:** nnU-Net preprocessing and network configuration.

Parameter	Setting
Input image	RGB laparoscopic frame, 1,920 × 1,080 pixels
Sampling rate	1 frame/s
R-channel preprocessing	95th-percentile clipping: 72–255; mean 163.3; SD 40.7
G-channel preprocessing	95th-percentile clipping: 28–244; mean 104.5; SD 41.1
B-channel preprocessing	95th-percentile clipping: 23–231; mean 88.9; SD 38.4
Prediction patch	896 × 1,792 pixels
Kernel	2D, 3 × 3
Batch size	2
U-Net levels	9
Feature maps	32, 64, 128, 256, 512, 512, 512, 512, 512
Convolutions per level	2
Training duration	1,000 epochs
Inference hardware	NVIDIA RTX 3090, 24 GB

The exported configuration specifies a frame-wise 2D implementation; it does not document temporal 3D convolution.

### Image annotation and reference standard

**FIGURE 2:**
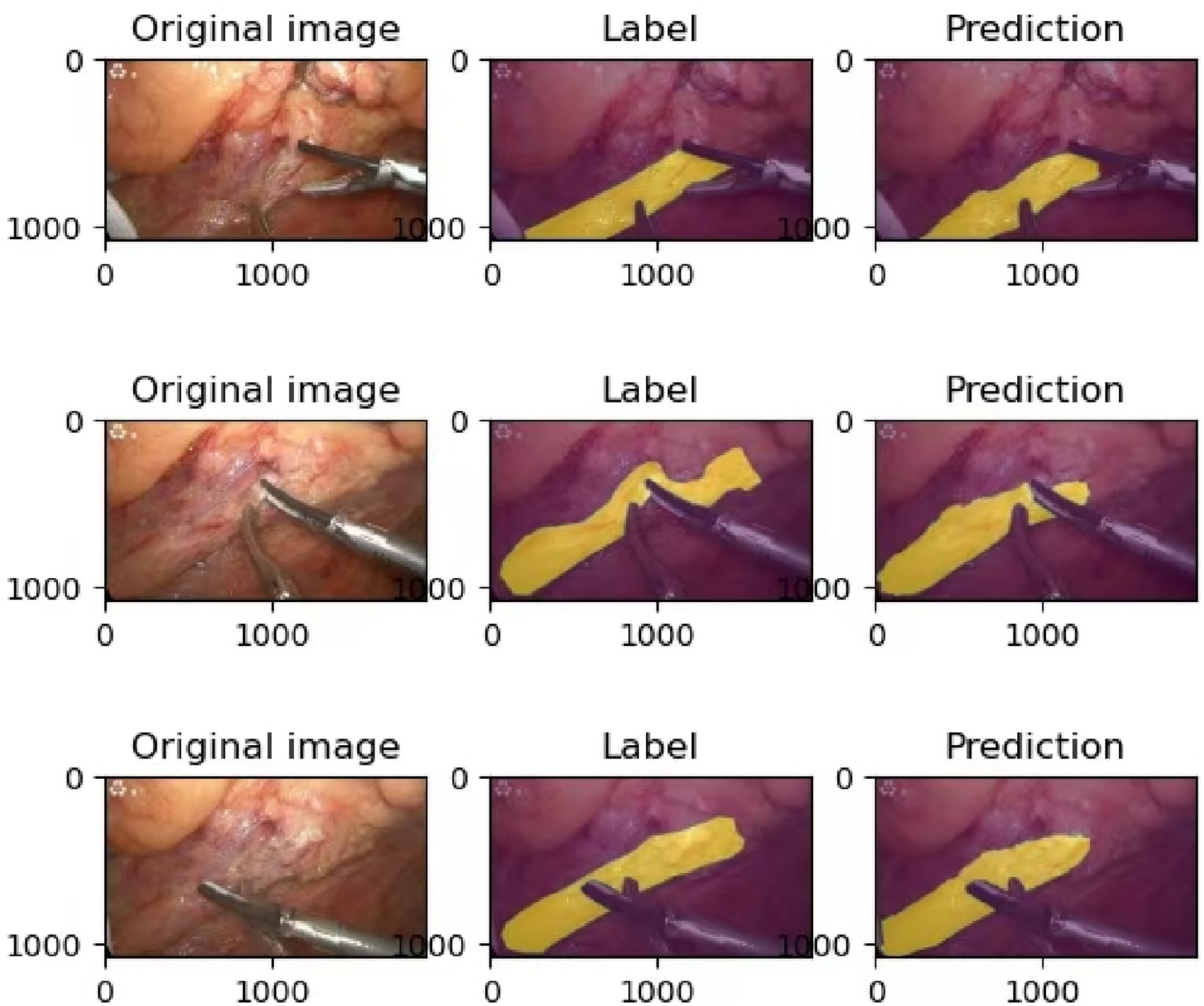
Representative example of IMA recognition. From left to right: the original laparoscopic image, the manually annotated ground truth, and the model-predicted segmentation.

Two senior gastrointestinal surgeons, each with more than 8 years of independent experience in LRR, annotated vessel contours separately using the polygon tool in LabelMe. Fine boundaries were traced at the pixel level. Because the operative field could contain fascial reflections, fat coverage, adhesions, and anatomical variation, annotation generally required 60–90 s per image and more than 2 min in some locally adherent cases. The IMA was identified by confirming its root at the division phase and tracing the vascular pedicle backward through consecutive frames. The IMV was followed from initial mobilization to complete division. A representative comparison of the original laparoscopic image, the manually annotated ground truth, and the model-predicted IMA segmentation is shown in Figure 2.

A differential rule was used for occlusion. When a vessel was only partly obscured by an instrument or a thin layer of fat, the hidden boundary was interpolated from visible wall tangents, anatomical knowledge, and neighboring frames to maintain a coherent contour. When the vessel was completely obscured by gauze, a suction device, or an opaque organ and could not be identified reliably, the frame was excluded. This strategy was intended to restrict supervision to regions that could be observed with reasonable confidence and to avoid introducing labels based primarily on speculation.

To reduce subjective bias, the study used independent dual annotation with blinded third-expert adjudication. The Dice similarity coefficient (DSC) between the two masks was calculated for each image. When DSC was at least 0.85, the pixelwise mean of the intersection and union masks was used as the final reference. Images with DSC below 0.85 were reviewed by a third senior surgeon with more than 15 years of primary-operator experience in LRR. The adjudicator examined the corresponding 30-frames/s video segment, vascular anatomy, and temporal context before defining the final mask. Agreement was assessed in a random 10% sample (*n* = 272), with a Cohen kappa of 0.87, indicating high consistency.

### Model development and labeling strategies

The segmentation network was the automatically configured U-Net architecture generated by nnU-Net v2 (30, 31). The encoder-decoder comprised nine resolution levels, with 32, 64, 128, 256, and five consecutive 512-channel feature-map stages. Each level contained two two-dimensional 3 × 3 convolutions. The batch size was 2, and training continued for 1,000 epochs. Inference efficiency was measured on an NVIDIA RTX 3090 GPU with 24 GB of memory (Table 1).

Training used an equally weighted combination of Dice loss and cross-entropy loss (Equation 3). Dice loss optimized regional overlap between the prediction and reference mask (Equation 1), whereas cross-entropy penalized pixel-level misclassification (Equation 2). Three labeling strategies were evaluated: one three-class model distinguishing IMA, IMV, and background; one binary IMA-versus-background model; and one binary IMV-versus-other model. This comparison represented a limited ablation of label organization and was not intended to replace comprehensive ablation of loss functions, preprocessing, augmentation, patch size, or network architecture (Table 2).


$$D\,i\,c\,e_{l\,o\,s\,s}=1-D\,i\,c\,e=1-\frac{2\,T\,P}{2\,T\,P+F\,P+F\,N}$$ (1)

**TABLE 2:** Labeling strategies used for model training

Strategy	Labels	Purpose
Three-class	IMA / IMV / background	Single model distinguishes both vessels and background
Binary IMA	IMA / background	Determines whether each pixel belongs to the IMA
Binary IMV	IMV / other	Determines whether each pixel belongs to the IMV

*(where TP, FP, and FN denote true positives, false positives, and false negatives, respectively)*


$$C\,E_{l\,o\,s\,s}=-\frac{1}{N}\,\sum_{i=1}^{N}\left[y_{i}\cdot l\,o\,g\,\left(p_{i}\right)\,\left(1-y_{i}\right)\cdot l\,o\,g\,\left(1-p_{i}\right)\right]$$ (2)

$$T\,o\,t\,a\,l_{l\,o\,s\,s}=0.5\cdot D\,i\,c\,e_{l\,o\,s\,s}+0.5\cdot C\,E_{l\,o\,s\,s}$$ (3)

### Performance outcomes and surgeon evaluation

Consensus annotations served as the reference standard. Segmentation was evaluated using the Dice coefficient, precision, and recall. Dice was defined as 2TP/(2TP + FP + FN), precision as TP/(TP + FP), and recall as TP/(TP + FN). Frame rate, network processing time per frame, and peak GPU memory use were also recorded. Because frames from the same patient were correlated, thousands of frames were not treated as independent observations for inflated significance testing; comparisons between labeling strategies were interpreted descriptively.

Preliminary usability was assessed by 20 practicing gastrointestinal surgeons who had not participated in training, annotation, or test-set adjudication. Participants were grouped by experience as junior (<5 years, *n* = 7), intermediate (5–10 years, *n* = 7), or expert (>10 years, *n* = 6). Each surgeon independently viewed 10 randomly ordered short videos containing segmentation overlays and scored three items on a five-level Likert scale from 0 to 4: Q1, perceived accuracy of vessel position and contour; Q2, acceptability of false-positive or missed detections; and Q3, future potential as an intraoperative recognition aid (Table 3). Model Dice, precision, and recall were not disclosed before rating. Scores were averaged across the 10 videos for each surgeon.

**TABLE 3:** Preliminary surgeon-rating questionnaire.

Question	0	1	2	3	4
Q1. Perceived accuracy of IMA/IMV overlay	Very poor	Poor	Fair	Good	Very good
Q2. Acceptability of recognition errors	Completely unacceptable	Mostly unacceptable	Neutral	Acceptable	Highly acceptable
Q3. Potential value as an intraoperative adjunct	None	Low	Moderate	High	Very high

Scores ranged from 0 to 4. This questionnaire assesses perceived feasibility and acceptability, not clinical effectiveness.

Exploratory comparisons among experience groups used the Kruskal-Wallis test because of the small, unequal groups and ordinal source ratings. Inter-rater reliability was assessed with a two-way random-effects, absolute-agreement intraclass correlation coefficient (ICC). ICC(2,1) represented the reliability of one surgeon, and ICC(2,k) represented the reliability of the mean across all 20 surgeons. Point estimates and 95% confidence intervals (CIs) were reported; CIs were obtained by percentile bootstrap resampling at the video level. ICC values below 0.50, 0.50–0.75, 0.75–0.90, and above 0.90 were interpreted as poor, moderate, good, and excellent reliability, respectively (32). The ICC analysis treated 10 videos as targets and 20 surgeons as raters. Because only 10 videos were assessed, wide bootstrap CIs were expected and were used to describe uncertainty rather than establish general reliability across all operative conditions. Analyses were performed using Python 3.9.23 (Python Software Foundation, Wilmington, DE, USA) and R 4.5.1 (R Foundation for Statistical Computing, Vienna, Austria), with RStudio as the integrated development environment.

## Results

### Patient characteristics

The median patient age was 66.5 years (range, 37–75), and 12 of 16 patients (75.0%) were men. Median body mass index was 23.8 kg/m^2^ (range, 19.3–30.1). According to the eighth edition of the Union for International Cancer Control staging system, 10 patients had stage II disease and 6 had stage III disease. Median intraoperative blood loss was 50 mL (range, 35–60), and median operating time was 175 min (range, 145–220). These perioperative variables describe the source cohort only and cannot be used to infer an effect of AI assistance (Table 4).

**TABLE 4:** Patient characteristics.

Characteristic	*N* = 16
Age, years	66.5 (37–75)
Male sex	12 (75.0%)
BMI, kg/m^2^	23.8 (19.3–30.1)
UICC stage II	10 (62.5%)
UICC stage III	6 (37.5%)
Blood loss, mL	50 (35–60)
Operative time, min	175 (145–220)

Continuous variables are median (range). BMI, body mass index; UICC, Union for International Cancer Control.

### Segmentation performance and inference efficiency

In the independent internal holdout test cohort, vessel-specific binary models provided the most balanced performance. For the IMA, Dice, precision, and recall were 0.940 +/− 0.025, 0.945 +/− 0.023, and 0.940 +/− 0.020, respectively. For the IMV, the corresponding values were 0.980 +/− 0.010, 0.982 +/− 0.017, and 0.978 +/− 0.018. In the three-class model, test-set Dice values were 0.940 +/− 0.019 for the IMA and 0.962 +/− 0.021 for the IMV. The principal benefit of binary labeling was therefore observed for IMV segmentation, whereas IMA Dice was essentially unchanged between strategies (Table 5).

**TABLE 5:** Segmentation performance by labeling strategy.

Strategy	Metric	Train IMA	Train IMV	Test IMA	Test IMV
Three-class	Dice	0.945 ± 0.018	0.965 ± 0.016	0.940 ± 0.019	0.962 ± 0.021
Three-class	Precision	0.920 ± 0.020	0.960 ± 0.015	0.920 ± 0.026	0.955 ± 0.029
Three-class	Recall	0.971 ± 0.011	0.970 ± 0.012	0.961 ± 0.024	0.969 ± 0.028
Binary IMA	Dice	0.953 ± 0.023	–	0.940 ± 0.025	–
Binary IMA	Precision	0.940 ± 0.020	–	0.945 ± 0.023	–
Binary IMA	Recall	0.966 ± 0.018	–	0.940 ± 0.020	–
Binary IMV	Dice	–	0.983 ± 0.011	–	0.980 ± 0.010
Binary IMV	Precision	–	0.985 ± 0.009	–	0.982 ± 0.017
Binary IMV	Recall	–	0.981 ± 0.009	–	0.978 ± 0.018

Values are mean ± SD. The test cohort was an internal patient-level holdout set of three patients and was not an external validation cohort.

The model processed test video at 12.7 frames/s. Network processing required approximately 0.08 s per frame, and peak GPU memory use was approximately 12.6 GB. This throughput supported responsive near-real-time visualization but remained below the 25–30 frames/s commonly used by laparoscopic video systems. A representative model-output video illustrating automatic IMA recognition during LRR is provided as Supplementary Video 1.

### Surgeon ratings

Overall scores were 3.41 +/− 0.09 for perceived recognition accuracy (Q1), 3.22 +/− 0.08 for output acceptability (Q2), and 3.39 +/− 0.09 for clinical application potential (Q3). Exploratory comparisons among junior, intermediate, and expert surgeons were not statistically significant (*P* = 0.068, 0.117, and 0.144, respectively) (Table 6). The ICC(2,1) values for Q1, Q2, and Q3 were 0.806 (95% CI, 0.49–1.00), 0.757 (95% CI, 0.31–0.98), and 0.782 (95% CI, 0.33–1.00), indicating good single-rater agreement. The corresponding ICC(2,k) values were 0.988, 0.984, and 0.986 (Table 7). The wide CIs were primarily attributable to evaluation of only 10 videos. Each experience group included only six or seven surgeons; absence of a statistically significant difference should not be interpreted as equivalence across experience levels.

**TABLE 6:** Preliminary ratings by surgeon experience.

Item	Junior *n* = 7	Intermediate *n* = 7	Expert *n* = 6	Overall *n* = 20	*P* value
Q1. Perceived accuracy	3.39 ± 0.09	3.37 ± 0.08	3.48 ± 0.08	3.41 ± 0.09	0.068
Q2. Acceptability	3.17 ± 0.10	3.23 ± 0.08	3.27 ± 0.05	3.22 ± 0.08	0.117
Q3. Future potential	3.44 ± 0.08	3.37 ± 0.10	3.35 ± 0.08	3.39 ± 0.09	0.144

Scores range from 0 to 4. *P* values are from exploratory Kruskal–Wallis tests.

**TABLE 7:** Inter-rater reliability of preliminary surgeon ratings.

Item	Model	Estimate	Bootstrap 95% CI	Interpretation
Q1. Perceived accuracy	ICC (2,1)	0.806	0.49–1.00	Good
Q2. Acceptability	ICC (2,1)	0.757	0.31–0.98	Good
Q3. Future potential	ICC (2,1)	0.782	0.33–1.00	Good
Q1. Perceived accuracy	ICC (2,k)	0.988	0.95–1.00	Excellent
Q2. Acceptability	ICC (2,k)	0.984	0.90–1.00	Excellent
Q3. Future potential	ICC (2,k)	0.986	0.91–1.00	Excellent

A two-way random-effects, absolute-agreement model was used. ICC(2,1) indicates single-rater reliability; ICC(2,k) indicates reliability of the mean rating across 20 surgeons.

## Discussion

This proof-of-concept study showed that an nnU-Net-configured U-Net could segment the IMA and IMV with high accuracy in selected white-light laparoscopic images while operating at a near-real-time speed. Vessel-specific binary labeling produced the clearest improvement for the IMV. A plausible explanation is that the IMA and IMV are usually exposed during different stages of LRR, so a three-class model receives limited frames in which both vessels provide useful simultaneous context. Nevertheless, the cohort included only 16 patients, the holdout test set contained only 3 patients, and no external validation was performed. The results should therefore be interpreted as early evidence of technical feasibility rather than proof of clinical benefit.

Differences between IMA and IMV performance may reflect both anatomy and data composition. The IMV is often exposed as a relatively isolated, slender structure against a comparatively homogeneous mesenteric background. In contrast, the IMA origin may be surrounded by variable fat, lymphatic tissue, autonomic nerves, and branching vessels. Conversely, the exceptionally high IMV Dice score may indicate that the holdout videos contained favorable views. Larger studies should report performance by vessel, exposure phase, body mass index, and operative-field difficulty rather than relying only on an overall average.

The IMA origin and mesorectal fascial planes are established landmarks in TME (33–36). The most clinically meaningful role for a recognition system would not be to color a vessel only after complete dissection, but to amplify visual cues during the earlier stages of tissue handling. We traced vessel identity backward from clearly exposed or divided segments using consecutive video frames, which allowed partially exposed vessels to be included. Standard white-light laparoscopy, however, cannot visualize vessels hidden completely by thick fat, gauze, instruments, blood, or organs. The appropriate role of the model is therefore to enhance visible anatomy, not to infer invisible structures or replace preoperative imaging and surgical judgment.

This role is complementary to patient-specific three-dimensional vascular reconstruction. Preoperative CT angiography can reveal variations of the IMA and left colic artery and assist procedural planning (14), but pneumoperitoneum and tissue traction limit direct correspondence between a static reconstruction and the live laparoscopic view. A future multimodal system could register a preoperative vascular map to intraoperative video segmentation while incorporating both vascular-variant priors and the current visual field. Such registration should be treated as an additional source of probabilistic information rather than as a fixed anatomical truth.

Previous studies have reported automated segmentation of the IMA, right-sided colonic vessels, prostate, dissection planes, and other intraoperative structures (18–28, 37). Kitaguchi et al. reported an IMA Dice score of approximately 0.816 (28), below the internal value observed here. Direct numerical comparison is not justified, however, because studies differ in case mix, imaging systems, annotation phase, frame selection, and data partitioning. The high scores in our study may partly reflect single-center homogeneity, consistent equipment, and images in which recognizable vascular cues were already present. External testing on unedited procedures from different surgeons and systems is essential to quantify the reduction in performance expected under domain shift.

The present system was a frame-wise two-dimensional U-Net. Sampling at 1 frame/s reduced redundancy but did not constitute temporal modeling. Before claiming that temporal information improves performance, the current baseline should be compared directly with true three-dimensional U-Net variants (38), convolutional recurrent networks, video Transformers, temporal state-space models, or optical-flow-guided segmentation. For small medical datasets, U-Net remains a reasonable baseline because its architecture is transparent, parameter-efficient, and reproducibly configured through nnU-Net (30, 31). Nonetheless, modern architectures should be benchmarked on exactly the same patient-level splits and training budget.

Recent studies have explored multiscale convolution, attention, boundary refinement, hierarchical Transformers, contrastive learning, knowledge distillation, generative augmentation, and ensemble learning in segmentation and classification tasks (39–50). TrionixNet and TATIMPA are closer to the present task because they are segmentation models, whereas several other cited approaches address classification or provide reviews (40–47). Their reported accuracy or area under the curve cannot be transferred directly to laparoscopic vessel segmentation. Conceptually, multibranch features may help distinguish subtle vessel-wall texture, windowed attention may combine local detail with broader anatomical context, and contrastive pretraining or distillation may stabilize learning from limited data. Ensemble methods may reduce model-specific bias but may also increase latency and memory use.

A fair future benchmark should include 2D U-Net/nnU-Net, temporal or 3D U-Net, Swin-Unet, TransUNet, SegFormer, medical Segment Anything Model variants, vascular Mamba models, and lightweight YOLO segmentation variants (39, 48–52). All models should use identical patient-level partitions, input resolution, augmentation, optimization budget, and hardware. Evaluation should include not only Dice, intersection over union, precision, and recall, but also boundary F1, 95th-percentile Hausdorff distance, mean surface distance, small-branch recall, vascular continuity, frame-to-frame jitter, duration of persistent missed detection, parameter count, floating-point operations, peak memory, and end-to-end latency. Prespecified difficult-scene subgroups should include bleeding, smoke, glare, motion blur, instrument occlusion, and rapid camera movement.

Segment Anything Model workflows require particular scrutiny. MedSAM benefits from large-scale medical pretraining but is prompt based, and branching vascular structures remain challenging (51). Manually drawing a prompt box during surgery would interrupt workflow, whereas automatic prompting adds a detection stage and creates another pathway for error propagation. Comparisons should distinguish fully automatic fine-tuning from interactive prompting and include prompt-generation time in latency estimates. Mamba-based models offer near-linear modeling of long-range dependencies, but existing vessel studies largely involve retinal, abdominal, or cerebral images rather than white-light laparoscopic video (52). Greater architectural complexity alone should not be assumed to yield superior clinical performance.

The comparison between three-class and binary labeling was a limited ablation and cannot isolate the contributions of Dice loss, cross-entropy, channel preprocessing, augmentation, sliding-window size, or nnU-Net self-configuration. Conducting many combinations in only 16 patients would generate unstable, data-dependent conclusions. Subsequent work should prespecify a small number of clinically and technically meaningful variants and use patient-level resampling rather than treating correlated frames as independent samples.

Throughput of 12.7 frames/s was responsive in an offline demonstration but remained below the native rate of most laparoscopic systems. When overlay updates are slower than video acquisition, contours may lag or appear to jump during fine dissection. The reported 0.08 s represented network processing only, not full latency from video capture to display. Clinical deployment requires measurement of acquisition, preprocessing, inference, postprocessing, and rendering as one end-to-end pipeline. Mixed-precision inference, pruning, quantization, TensorRT optimization, asynchronous pipelines, and lighter encoders may improve throughput, but any acceleration must be evaluated for loss of small-branch sensitivity and boundary stability.

Annotation policy also influences apparent performance. Restricting labels to reliably visible anatomy reduces noise from subjective inference, but excluding fully occluded vessels enriches the dataset for scenes in which visual cues are already available and may overestimate performance in difficult conditions. Future datasets should prospectively record the degree of occlusion, bleeding, smoke, glare, and motion, as well as annotation and adjudication time. Training may benefit from occlusion augmentation, synthetic smoke, illumination perturbation, Cutout, Copy-Paste, hard-example mining, semi-supervised learning, active learning, and uncertainty estimation. During inference, low-confidence overlays should be hidden rather than persistently displaying a potentially misleading contour.

The surgeon questionnaire assessed preliminary acceptance only. Viewing selected short clips with an overlay does not reproduce time pressure, instrument manipulation, hand-eye coordination, or competing visual demands during an operation. There was no non-AI control condition and no objective measure of task efficiency or cognitive workload. ICC quantified agreement among raters for these 10 videos, not clinical effectiveness. The very high ICC(2,k) values largely reflect averaging across 20 raters, which reduces individual variability; the more conservative ICC(2,1) estimates were 0.757–0.806 and had wide CIs. Agreement, correctness of ratings, and clinical validity are distinct concepts.

The next human-factors study should use a randomized crossover video-simulation design in which surgeons at different experience levels identify the IMA and IMV with and without AI support. Primary outcomes should include time to vessel recognition and the rate of first anatomical misidentification. Mixed-effects models could account for surgeon, video difficulty, study period, and randomized order. The NASA Task Load Index could quantify mental demand, temporal pressure, and effort (53). Such simulation can test recognition efficiency and cognitive workload, but it cannot establish that the model reduces intraoperative bleeding in patients.

Clinical evaluation should proceed in stages. A prospective silent-deployment phase should first record performance and failure modes on complete, unedited procedures without displaying predictions to the operating surgeon. Controlled human-AI collaboration studies could then assess recognition time, response to incorrect prompts, and task completion. Only after these steps would studies of patient-level outcomes be appropriate, including unintended vascular injury, unplanned hemostatic intervention, blood loss, operating time, conversion to open surgery, lymphadenectomy quality, and perioperative complications.

Regulatory and ethical requirements are integral to translation. A system used for intraoperative guidance may qualify as software as a medical device or as an embedded medical-device function. The surgeon must retain final decision authority. The interface should prioritize the original video, permit immediate deactivation, suppress low-confidence output, and warn of latency or signal loss. Data minimization, de-identification, access control, audit logs, model versioning, and drift monitoring are also required. These safeguards align with the DECIDE-AI emphasis on human factors, failure modes, and staged clinical assessment (29).

The study has several major limitations: its retrospective single-center design and small sample; an internal holdout test set of only three patients; absence of cross-institution, cross-surgeon, and cross-device validation; use of selected frames rather than complete unedited videos; lack of dedicated temporal modeling; no comprehensive ablation or direct benchmark against major contemporary architectures; measurement of network time rather than end-to-end latency; inability to generate supervised labels for completely invisible vessels; and a subjective surgeon survey that cannot demonstrate improved task performance or patient outcomes. Blood loss and operating time were descriptive cohort characteristics and cannot be attributed to AI assistance.

## Conclusion

An nnU-Net-configured two-dimensional U-Net achieved high internal test-set segmentation scores for the IMA and IMV in selected laparoscopic images and operated at a near-real-time speed. The study demonstrates technical feasibility, not clinical effectiveness. Multicenter external validation, difficult-scene testing, standardized benchmarking, end-to-end acceleration, objective human-factors experiments, and prospective clinical studies are necessary before the system can be considered a reliable intraoperative decision-support tool.

## Data Availability

The datasets used and/or analyzed during the current study are available from the corresponding author on reasonable request.

## Supplementary material

Supplementary Video 1 Representative model-output video illustrating automatic recognition and segmentation of the inferior mesenteric artery during laparoscopic rectal resection.

Supplementary Data Sheet 1 Completed 2022 DECIDE-AI reporting checklist.
